# Effectiveness of Thymol-Loaded Chitosan Nanoparticles Against ***Toxocara Vitulorum* Infective Larvae In Vitro**

**DOI:** 10.1007/s11686-025-01196-8

**Published:** 2026-01-13

**Authors:** Sara Bayoumi Ali, Ayman Saber Mohamed, Sohair R. Fahmy, Fatma Mahmoud Mohammed, Mona F. Khalil

**Affiliations:** 1 Zoology Department, Faculty of Science, Cairo University, Giza, 12613 Egypt; 2 Department of Zoology and Entomology, Faculty of Science, Helwan University, Cairo, Egypt

**Keywords:** *Toxocara vitulorum*, Larvae, Thymol, Chitosan, Nanoparticles

## Abstract

**Purpose:**

This study assessed the impact of different concentrations of thymol-loaded chitosan nanoparticles (TC NPs) on the physiological condition and surface morphology of *Toxocara vitulorum* infective larvae in vitro.

**Methods:**

Thymol-loaded chitosan nanoparticles were produced utilizing the emulsion-ionic gelation process with sodium tripolyphosphate and then freeze dried. UV-Vis, XRD, TEM, and DLS were used to analyze optical, structural, and size properties, as well as encapsulation efficiency and loading capacity. Toxocara vitulorum worms were gathered from buffaloes, and female worms were employed to extract and hatch eggs in the laboratory. The larvae were exposed to different concentrations of thymol, chitosan NPs, and TC NPs (0, 1000, 2000, 3000, 4000, and 5000 µg/mL) and were kept at 37 °C for 24 h Trypan blue staining and scanning electron microscopy were used to assess the toxicity and larvicidal activity of TC NPs at various doses. The oxidative stress indicators (GSH, MDA, CAT, and NO) were evaluated in treated larvae.

**Results:**

The exposed larvae to TC NPs had an increase in malondialdehyde, catalase and nitric oxide levels, while a depletion in glutathione concentration. Light microscopy analysis indicated that the exposed larvae lost their coiling habit, exhibiting many holes and wrinkles. Moreover, scanning electron microscopy revealed morphological changes in the larvae’s body wall, including numerous erosional and fissured regions, along with both small and large blebs resulting from exposure to TC NPs.

**Conclusion:**

TC NPs at environmentally relevant doses demonstrated considerable antihelminthic action against *Toxocara vitulorum* infective larvae, establishing a successful model for parasite control research.

**Graphical Abstract:**

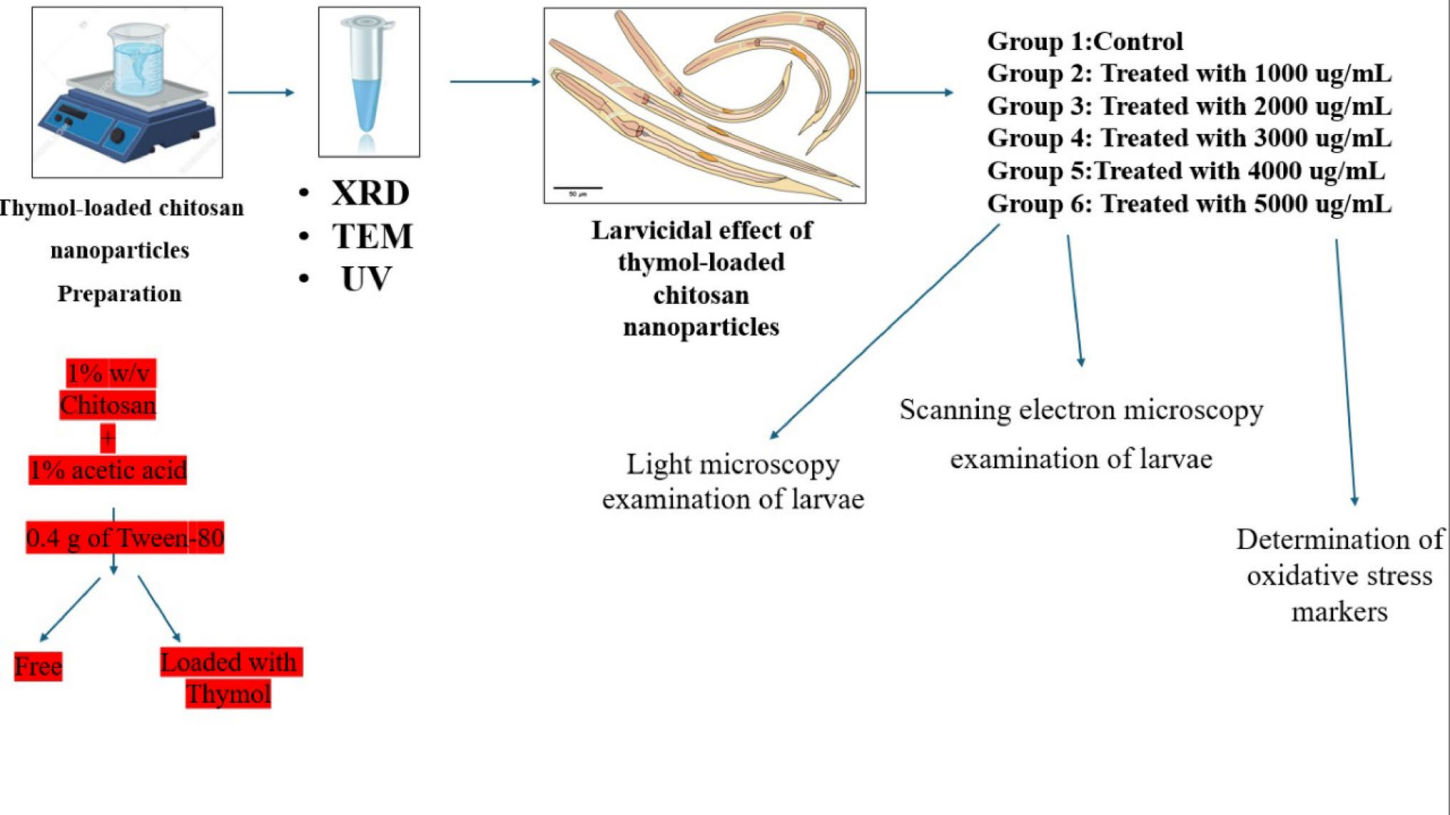

## Introduction

*Toxocara vitulorum* is a prevalent gastrointestinal nematode affecting buffaloes and cattle, particularly young calves [1]. The primary route of infection is through contamination of soil and water with *T. vitulorum* eggs obtained from the buffalo feces [2]. The larvae of *T. vitulorum* migrate via the bloodstream to multiple organs such as the liver, lungs, intestines and eyes, resulting in inflammation and injury [3–5]. Most larvae traverse the liver and lungs, resulting in poor weight gain, anorexia, diarrhea, constipation, dehydration, and unthriftiness in young calves [6]. The disease severity varies from asymptomatic to lethal infection [7]. The majority of anthelmintic agents employed in the management of parasitic worm infections interact with specific target proteins or modulate the electrical activity of neurons and muscles of the parasite [8]. This may result in paralysis, hunger, immunological response, and ejection of the worm [9]. However, contemporary anthelmintics have several limitations, including a restricted spectrum of activity against some parasitic species and the potential to induce drug resistance [10]. Consequently, nanomaterials present a promising therapeutic option for several parasite diseases affecting human and animal health, particularly in relation to the issue of drug resistance [11–13].

The field of nanotechnology pertains to the scientific and engineering disciplines operating at the nanoscale. Nanoparticles are usually referred to as particles less than 1000 nanometers [14]. In recent years, nanoparticles have garnered significant attention due to their distinctive qualities, including a high surface area to volume ratio, which can lead to unique chemical, physical, and biological characteristics [15].

There are a number of plant species that effectively combat various animal-borne parasites, and these plants show promise in controlling and halting the parasites’ life cycle [16–20]. Thyme, scientifically known as *Thymus vulgaris*, contains the active ingredient thymol [21]. The antimicrobial, antifungal, larvicidal, and acaricidal activities of thymol have been documented previously [22–26]. Thymol possesses biological actions such as antispasmodic, anti-inflammatory, and anti-carcinogenic properties [22, 27–30]. An alternative drug delivery system could be a viable alternative to increase thymol’s effectiveness, and one way to lessen these possible restrictions is to encapsulate the essential oils into suitable nanocarriers. Folle et al. [31] have shown that nanomaterials encapsulated with thymol are more soluble and have enhanced antiparasitic properties.

Biopolymer nanoparticles are considered effective drug delivery systems due to their ability to easily penetrate cell membranes and regulate, prolong, and precisely direct drug release [32]. Furthermore, they possess biodegradable, biocompatible, and non-toxic properties [32–34]. These special properties increased their efficacy against parasitic infections while reducing side effects, making them valuable in the treatment of parasites. Chitosan (CS), a partly deacetylated derivative of chitin, consists of D-glucosamine repeating units connected by β-(1–4) glycosidic linkages and has been extensively utilized as a drug delivery system in medicine [35–39]. The distinctive properties of chitosan nanoparticles (CNPs), due to their diminutive size and quantum size impact, may confer enhanced antibacterial efficacy. These are natural materials exhibiting exceptional physicochemical, antibacterial, and biological properties, rendering these nanoparticles eco-friendly materials with bioactivity that is highly safe for people [40, 41]. Therefore, the present study sought to assess the anthelmintic efficacy of thymol-loaded chitosan nanoparticles (TC NPs) against *Toxocara vitulorum* infective larvae.

## Materials and Methods

### Preparation of TC NPs

A 40 mL chitosan solution (1% [w/v]) was prepared by dissolving the chitosan particles in an aqueous solution of glacial acetic acid (1% [v/v]) and stirring overnight at ambient temperature (25 °C). Subsequently, a homogeneous mixture was achieved by adding 0.4 g of Tween-80 (HLB15.9) to the prepared chitosan solution and stirring at 60 °C for 2 h. To establish the oil phase of the emulsion, thymol (equivalent in weight to chitosan) was dissolved in CH _2_ Cl _2_ (4 mL). The oil phase of thymol was introduced into the agitated mixture in a dropwise manner to create an oil-in-water emulsion with a mass ratio of chitosan to thymol of 1:1. Lastly, the ion gelation of chitosan was induced by the gradual addition of the sodium triphosphate solution (0.5% [w/v]) to the oil-in-water emulsion in a dropwise way. The stirring persisted for 45 min. The chitosan nanoparticles that were produced were separated and collected by centrifugation at 979 g for 20 min. Subsequently, they were washed with deionized water multiple times to eliminate any unbound thymol. The prepared dispersions were subsequently freeze-dried promptly at − 40 °C for 12 h and stored at − 30 °C using a freeze dryer (FD5–2.5, China) [42].

### Characterization of TC NPs

####  Ultraviolet–Visible Spectroscopy

The absorption spectra of synthesized nanoparticles were used to observe the optical properties of TC NPs. At ambient temperature, the Varian Cary 5000 UV–visible spectrophotometer was employed to measure UV–Vis spectra with a wavelength spanning 200–800 nm.

#### X-Ray Diffraction (XRD) Analysis

The size and structural properties of TC NPs were analysed using XRD with Cu–Kα radiation (XPERT–PRO, PANAnalytical). Scherrer’s formula was employed to ascertain the crystallite size of the particles: K/β cosδ = d. In this configuration, K represents the crystallite form factor (0.89), λ is the X-ray wavelength of the Cu Kα radiation (0.154 nm), θ is the Bragg diffraction angle, and β is the full width at half maximum of the corresponding diffraction peak.

#### Transmission Electron Microscopy (TEM)

At the Electron Microscopy Unit, Faculty of Agriculture, Cairo University, the size and morphology of TC NPs were ascertained using electron microscopy at an accelerated voltage of 120 kV. TC NPs were dissolved in absolute ethanol, sonicated for 15 min, and then loaded on a metal stub. Next, place the grid in the TEM’s vacuum chamber, orient the sample, and direct the high-energy electron beam through it. Finally, the magnification should be adjusted to provide a clear image of the nanoparticles on the screen for imaging (JEM-JEM 2100 F; JEOL Ltd, Tokyo, Japan) [21].

#### Encapsulation Efficiency (EE) and Loading Capacity (LC) Determination

The UV–Vis spectrophotometer was employed to quantify the concentration of thymol encapsulated in chitosan nanoparticles. In a nutshell, a 5 mL solution of 2 mol/L HCl was used to reflux chitosan nanoparticles (10 milligrams) loaded with thymol at 100 °C for 45 min. Following this, the mixture was centrifµged at 10,000 rpm for 10 min, and 2 mL of unadulterated ethanol was added. The thymol content of the supernatant was analyzed at a wavelength of 275 nm after it was collected. The standard curve (R2 = 0.999) was employed to determine the concentration of thymol in absolute ethanol throµgh the measurement of its absorbance. All measurements were collected three times (*n* = 3). The following Eqs. (1) and (2) were employed to assess the EE and LC of thymol, respectively:

1 $$ {\text{EE }}(\% ) = \frac{{{\text{The total amount of loaded thymol}}}}{{{\text{The initial amount of thymol }}}} $$

1 $$ {\text{LC }}(\% ) = \frac{{{\text{The total amount of loaded thymol}}}}{{{\text{Weight of freeze - dried nanoparticles}}}} $$

#### Particle Size Measurement and Zeta Potential

Dynamic and phase analysis light scattering were performed to determine the particle size at 25 °C using dynamic light scattering (DLS) analysis (Zeta sizer Nano ZS90, Malvern Instruments, UK). The particle size results obtained from DLS were expressed as the Z-average mean diameter and polydispersity index (PDI).

### Sample Collection

Freshly isolated Gastrointestinal tracts from the livestock animals, the buffaloes *Bubalus bubalis* were collected during the current study from the slaughterhouse. They were transported to the Parasitology laboratory, Zoology Department, Faculty of Science, Cairo University. The various organs were separated from each other and placed individually in shallow plastic jars. The contents of the gastrointestinal tract were put into separate plastic containers and will be examined for helminth parasites, followed by standard methods of Boomker et al. [43]. *T. vitulorum* was identified based on the morphology. The fresh worms are translucent and whitish in colour and have a cylindrical wall.

#### Collection of Worms

**Fig. 1:**
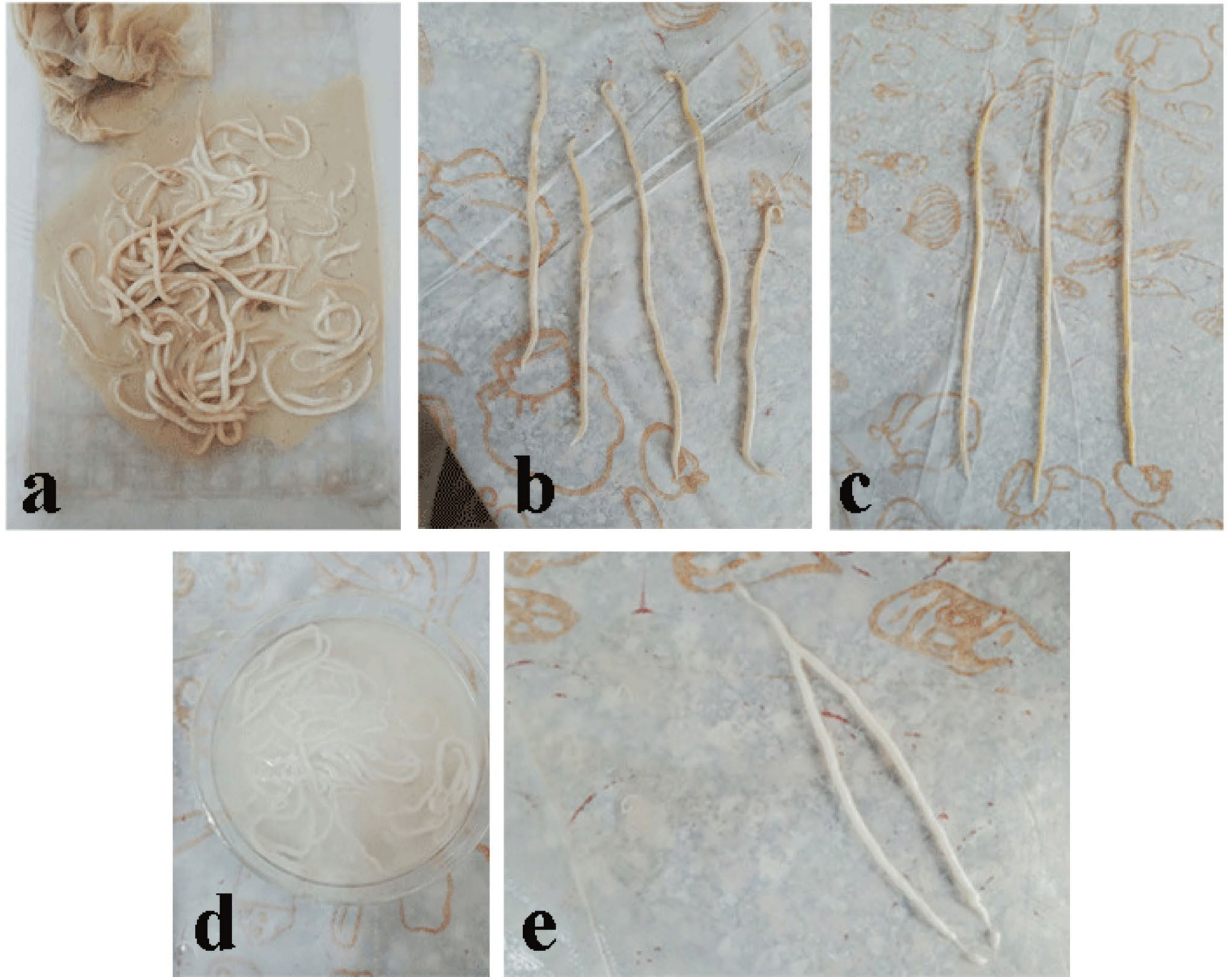
Collected adult *Toxocara vitulorum* preserved in fluid, showing elongated, cylindrical, cream-colored worms tangled together. **a** Freshly worms, **b** adult male worms, **c** adult female worms, **d** dissected uterus in acidic media, **e** isolated uterus of female worm

Adult female worms were separated and selected from male worms using two procedures. The first difference is in size and length, with female worms being longer and thicker than males. Second, by dissection, the uterus was found to be filled with eggs, which were examined under a light microscope. Following selection, the worms were rinsed in warm water from their gastrointestinal tracts before being moved to a flask containing Goodwin’s solution and kept at 37 °C (Fig. 1).

#### *In Vitro T. Vitulorum* Egg Embryonation

Ten female worms were dissected in acidified water (pH 3), the uteri removed, and cut to liberate the eggs. The retrieved eggs were centrifuged at 500 g for 5 min and utilized for embryonation and the development of the infective stage. Embryonation was conducted in a beaker containing 0.5% formalin, sealed with a hydrophobic cotton plug, and incubated at 36 °C for 20 days. The fluid containing the eggs was manually stirred twice daily to facilitate third-stage larvae development. After the incubation period, the embryonated eggs were rinsed three times in saline to eliminate the formalin solution [8].

#### Hatching Assay

The embryonated eggs were rinsed three times in normal saline using centrifugation at 2795 g for 5 min to remove the maintaining solution. The suspension that resulted was incubated at 37 °C in hatching media (4% NaOH, 4% NaOCl) for 24 h, then the infective eggs were centrifuged at 2795 g, and the supernatant was discarded. The pellet of eggs obtained was incubated in 0.2% Tween 20 for one hour, rinsed three times with saline, and hatched larvae were collected [44, 45].

#### Acute Toxicity Study

Different concentrations of TC NPs (31, 62, 125, 250, 500,1000, 2000,3000 µg/mL) were prepared to estimate their lethality. Fifty infective larvae in Goodwin’s solution were incubated with each concentration of TC NPs at 37 °C for 24 h in transparent containers along with the control group that had not been exposed to TCNPs [46].

#### Larvicidal Effect of Thymol-Loaded Chitosan Nanoparticles (TC NPs)

Four groups of larvae, i.e., control, thymol, chitosan NPs, and thymol-loaded chitosan NPs, were utilized. Ten mL of each treatment (1000, 2000, 3000, 4000, 5000 µg/mL) was added to one mL of nematode suspensions containing 50 freshly hatched larvae in a sterilized 6-well plastic tissue plate. In addition, 10 ml of Goodwin’s solution plus 1 mL of the nematode suspension serves as a control. The plates were kept at 37 °C for 24 h.

#### Light Microscopy Examination of Larvae

Following completion of the incubation, the larvae in the treatment solution for each concentration were centrifuged at 2795 g and the supernatant removed. The pellet was incubated with 2% trypan blue for 15 min, and the stained larvae were considered dead.

#### Scanning Electron Microscopy Examination of Larvae

The larvae subjected to various concentrations of TC NPs were preserved in 2.5% glutaraldehyde within a 0.1 M cacodylate buffer, subsequently rinsed in the same buffer at pH 7.2, post-fixed in a solution of 1% osmium tetroxide and 1.25% potassium ferrocyanide (pH 7.2) for one hour and dehydrated through a graded series of ethanol solutions (30–100%). The samples were subjected to a critical point drying process using Bomer-900, coated with gold-palladium in a Technics Hummer V, and subsequently analyzed with a Jeol SEM (Model JSM7610F, Jeol Ltd., Japan).

#### Oxidative Stress Assessment

The examined larvae were homogenized in phosphate-buffered saline and centrifuged at 2795 g. The supernatant obtained was used for the determination of reduced glutathione (GSH), malondialdehyde (MDA), catalase (CAT), and nitric oxide (NO) according to the instructions of the manufacturer, using Bio-Diagnostic kits (Giza Governorate, Egypt).

### Statistical Analysis

Statistical analysis was conducted using two-way analysis of variance (ANOVA) and the least significant difference (Duncan) post hoc test to compare group averages. Data are provided as mean ± SE, with *p* < 0.05 deemed significant.

## Results

### Characterization of TC NPs

#### Ultra-Violet–Visible Spectroscopy

**Fig. 2:**
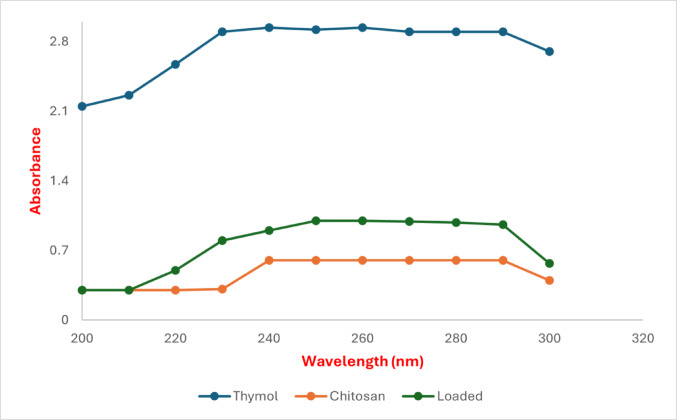
UV-Vis spectroscopy of thymol chitosan NPs and TC NPs

The formation of thymol, chitosan NPs and TC NPs was confirmed using UV-vis spectroscopy. The present examination of the UV spectra indicated that thymol extract had characteristic peaks between 230 and 290 nm. Whilst chitosan NPs had a distinctive peak between 240 and 290 nm. In addition, thymol-loaded chitosan nanoparticles had characteristic peaks between 230 and 290 nm (Fig. 2).

####  X-Ray Diffractometer (XRD) Analysis

**Fig. 3:**
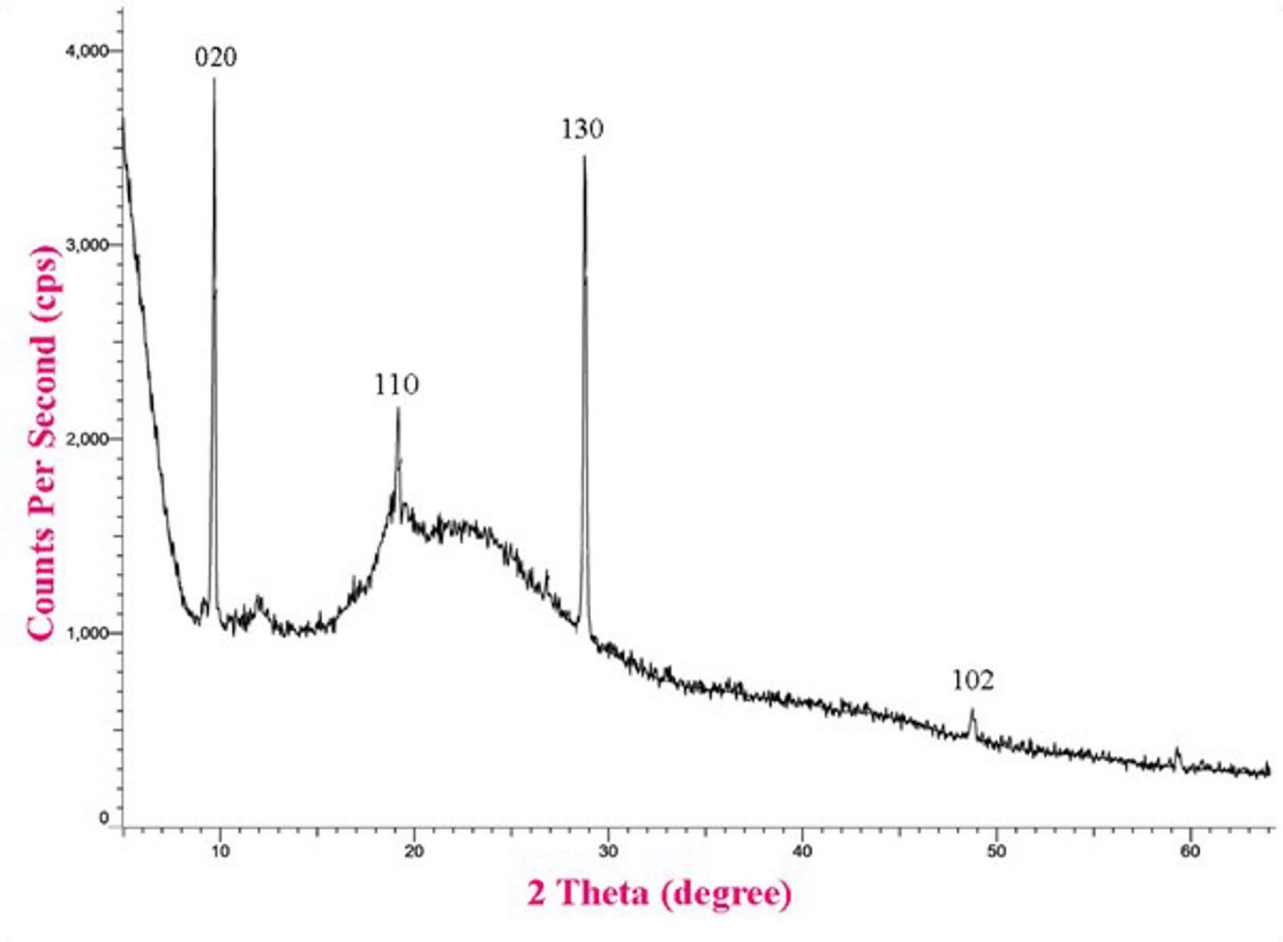
XRD Pattern of chitosan NPs

**Fig. 4:**
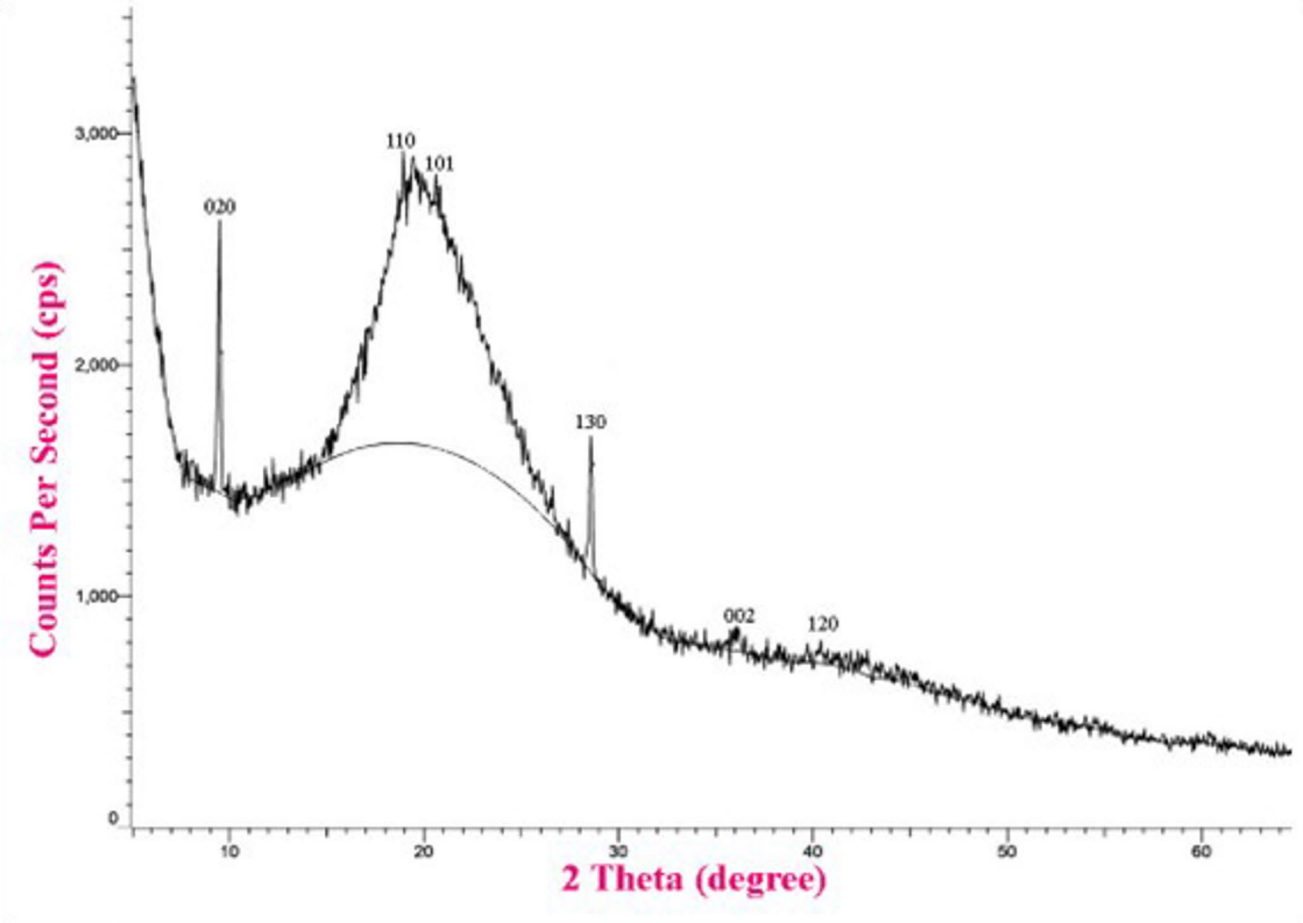
XRD Pattern of TC NPs

The structure of chitosan and TC NPs was determined using the XRD technique. The XRD peaks for chitosan NPs at 2 θ = 9.6°, 19.5°, 35.96° and 48.7° corresponded to the crystal planes 020, 110, 130 and 102. While the XRD peaks for TC NPS at 2 θ = 9.6°, 19.0°, 21.2°, 29.5°, 31.5°and 41° corresponded to the crystal plans 020, 110, 101, 130, 002 and 102. The average crystallite size, as determined using the Debye–Scherrer equation for chitosan NPs and TC NPs, was found to be approximately 135 nm and 140 nm, respectively (Figs. 3 and 4).

#### TEM Micrographs of TC NPs

**Fig. 5:**
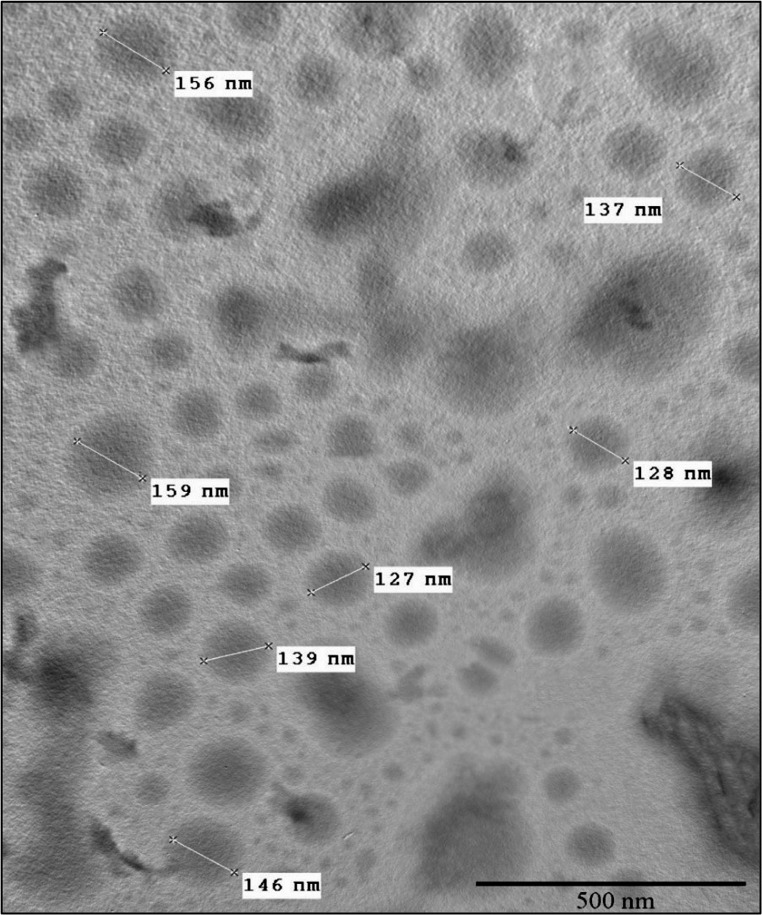
Transmission electron micrograph of TC NPs

TEM analysis showed that the surface of chitosan and TC NPs was spherical and smooth. The average particle size of chitosan NPs and TC NPs was approximately 141 ± 6 nm (*n* = 6) and 138.7 ± 4 nm (*n* = 6) (Fig. 5), respectively. It is important to mention that the Debye–Scherrer equation gets the crystallite size (i.e., the dimension of a single crystal domain) rather than the overall particle size. In contrast, the actual particulate size is provided by TEM analysis. Intriguingly, the crystallite sizes determined by XRD were exceedingly similar to the particle diameters observed by TEM (approximately 141 ± 6 nm and 138.7 ± 4 nm). This close agreement implies that the synthesized nanoparticles are primarily monocrystalline, with each particle consisting of a single crystal domain, rather than polycrystalline aggregates.

#### Encapsulation Efficiency (EE) and Loading Capacity (LC)

The EE of the thymol-loaded chitosan nanoparticles is approximately 96.6 ± 1.01%. Additionally, drug loading analysis revealed that the chitosan nanoparticles consistently maintained a loading capacity exceeding 50%, confirming their strong ability to retain and stabilize the incorporated drug over time.

#### Particle Dispersion Index and Zeta Potential

**Fig. 6:**
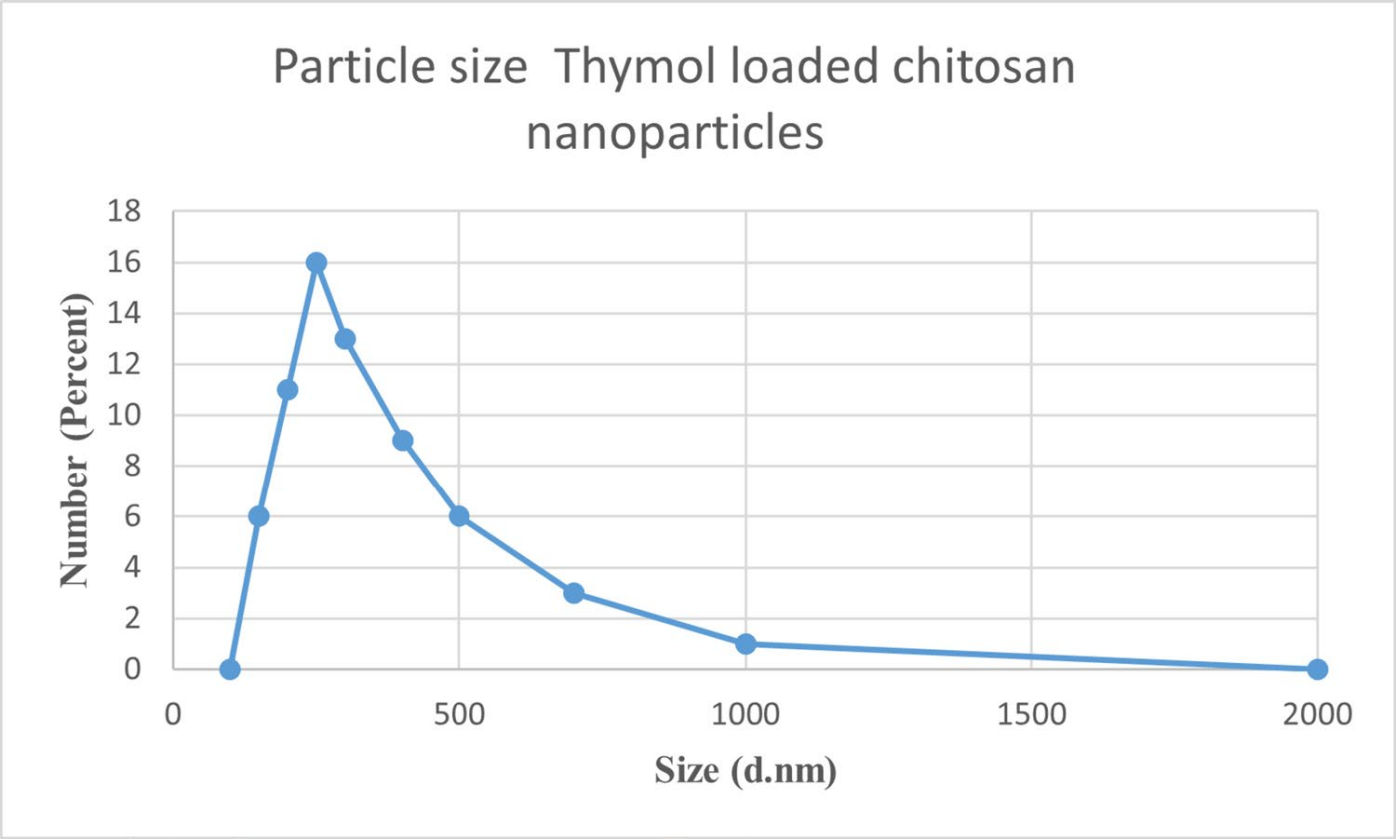
Particle size distribution of TC NPs

Dynamic light scattering (DLS) analysis revealed that the average particle sizes of chitosan nanoparticles and thymol-loaded chitosan nanoparticles were approximately 190 and 220 nm, respectively. Both formulations exhibited relatively narrow size distributions, as indicated by polydispersity index (PDI) values of 0.453 for chitosan nanoparticles and 0.513 for thymol-loaded chitosan (Fig. 6).

**Fig. 7:**
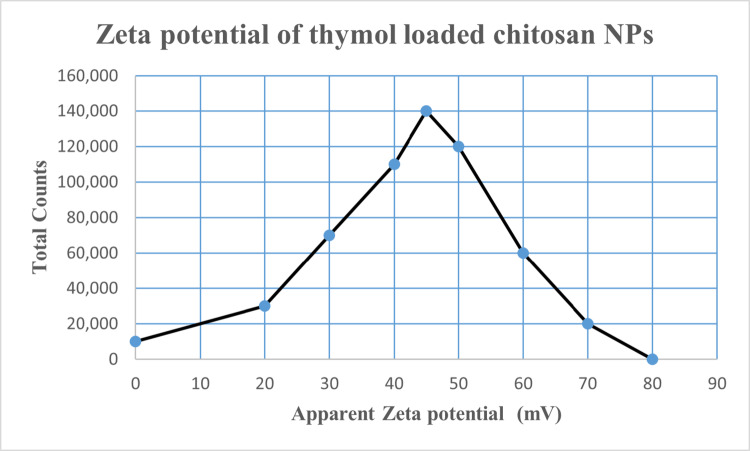
Zeta potential of TC NPs

The zeta potential analysis demonstrated that the chitosan nanoparticles had a zeta potential of + 51.9 ± 2.5 mV. On the other hand, the zeta potential of thymol-loaded chitosan nanoparticles was measured to be slightly lower, at + 42.4 ± 1.3 mV, as depicted in Fig. 7.

### Acute Toxicity Study

The larvae were subjected to chitosan and TC NPs (31, 62, 125, 250, 500, 1000, 2000, and 3000 µg/mL). At 3000 µg/mL of chitosan and TC NPs, half of the mortality was noted.

#### Larvicidal Effect of Thymol-Loaded Chitosan Nanoparticles (TC NPs)

##### Oxidative Stress Markers Assessment

Data shown in Table 1 demonstrated a significant increase (*p* < 0.05) in MDA, NO concentrations and CAT activity of *T. vitulorum* larvae exposed to thymol, chitosan NPs and TC NPs (1000, 2000, 3000, 4000, 5000 µg/mL) compared to the control group. In addition, there was a significant increase (*p* < 0.05) in the levels of GSH in *T. vitulorum* larvae exposed to thymol and TC NPs and a substantial decrease (*p* < 0.05) in the levels of GSH in *T. vitulorum* larvae exposed to chitosan NPs compared to the control group.

**Table 1:** Effectiveness of thymol-loaded Chitosan nanoparticles on oxidative stress markers of *Toxocara vitulorum* larvae

Groups	Concentrations (µg/ml)		Malondialdehyde (nM/g. protein)	Reduced glutathione (mM/g. protein)	Catalase (U/g. protein)	Nitric oxide (µM/g. protein)
Control	**0**		0.09 ± 0.00^a^	0.35 ± 0.02^e^	1.41 ± 0.02^a^	3.6 ± 0.10^a^
Thymol	**1000**		0.16 ± 0.00^b^	0.75 ± 0.02^h^	2.38 ± 0.14^f^	4.22 ± 0.10^b^
	**2000**		0.23 ± 0.01^d^	0.78 ± 0.01^i^	2.38 ± 0.15^f^	5.01 ± 0.05^c^
	**3000**		0.28 ± 0.00^e^	0.89 ± 0.05^j^	2.4 ± 0.30^f^	5.2 ± 0.11^d^
	**4000**		0.29 ± 0.01^f^	0.93 ± 0.05^k^	1.96 ± 0.10^c, d^	5.2 ± 0.13^d^
	**5000**		0.34 ± 0.00^g^	1.13 ± 0.00^l^	1.74 ± 0.01^b, c^	5.53 ± 0.05^e^
Chitosan NPs	**1000**		0.16 ± 0.00^b^	0.11 ± 0.00^d^	1.64 ± 0.11^a, b^	9.16 ± 0.13^f^
	**2000**		0.16 ± 0.00 ^b^	0.07 ± 0.01^c^	1.70 ± 0.12^b, c^	9.4 ± 0.16^g^
	**3000**		0.19 ± 0.00^c^	0.05 ± 0.00^b^	1.89 ± 0.08^b, c,d^	13.0 ± 0.11^k^
	**4000**		0.20 ± 0.01^c^	0.03 ± 0.00^a^	2.1 ± 0.18 ^d, e^	9.77 ± 0.13^h^
	**5000**		0.21 ± 0.01^c^	0.02 ± 0.00^a^	2.32 ± 0.05^e, f^	13.5 ± 0.17^l^
Thymol loaded chitosan NPs	**1000**		0.20 ± 0.01^c^	0.58 ± 0.01^g^	2.39 ± 0.01^f^	12.0 ± 0.23^i^
	**2000**		0.20 ± 0.01^c^	0.55 ± 0.01^f^	2.39 ± 0.04^f^	12.0 ± 0.11^i^
	**3000**		0.23 ± 0.01^d^	0.75 ± 0.01^h^	2.83 ± 0.07^g^	12.7 ± 0.25^j^
	**4000**		0.31 ± 0.01^f^	1.14 ± 0.02^l^	3.5 ± 0.20^h^	13.9 ± 0.18^m^
	**5000**		0.33 ± 0.01^g^	1.18 ± 0.02^m^	3.7 ± 0.12^i^	13.94 ± 0.07^m^
Two-way ANOVA	**Effect of each treatment**		*p* < 0.05			
	**Effect of concentrations**		*p* < 0.05			
	**Interaction between treatment and concentrations**		*p* < 0.05			

Bold values indicate statistically significant differences (*p* < 0.05)

Values are means ± SEM (*N* = 8 per group)

Each value not sharing a common letter superscript is significantly different (*p* < 0.05)

### Light Microscopy Examination of *T. Vitulorum* Larvae

**Fig. 8:**
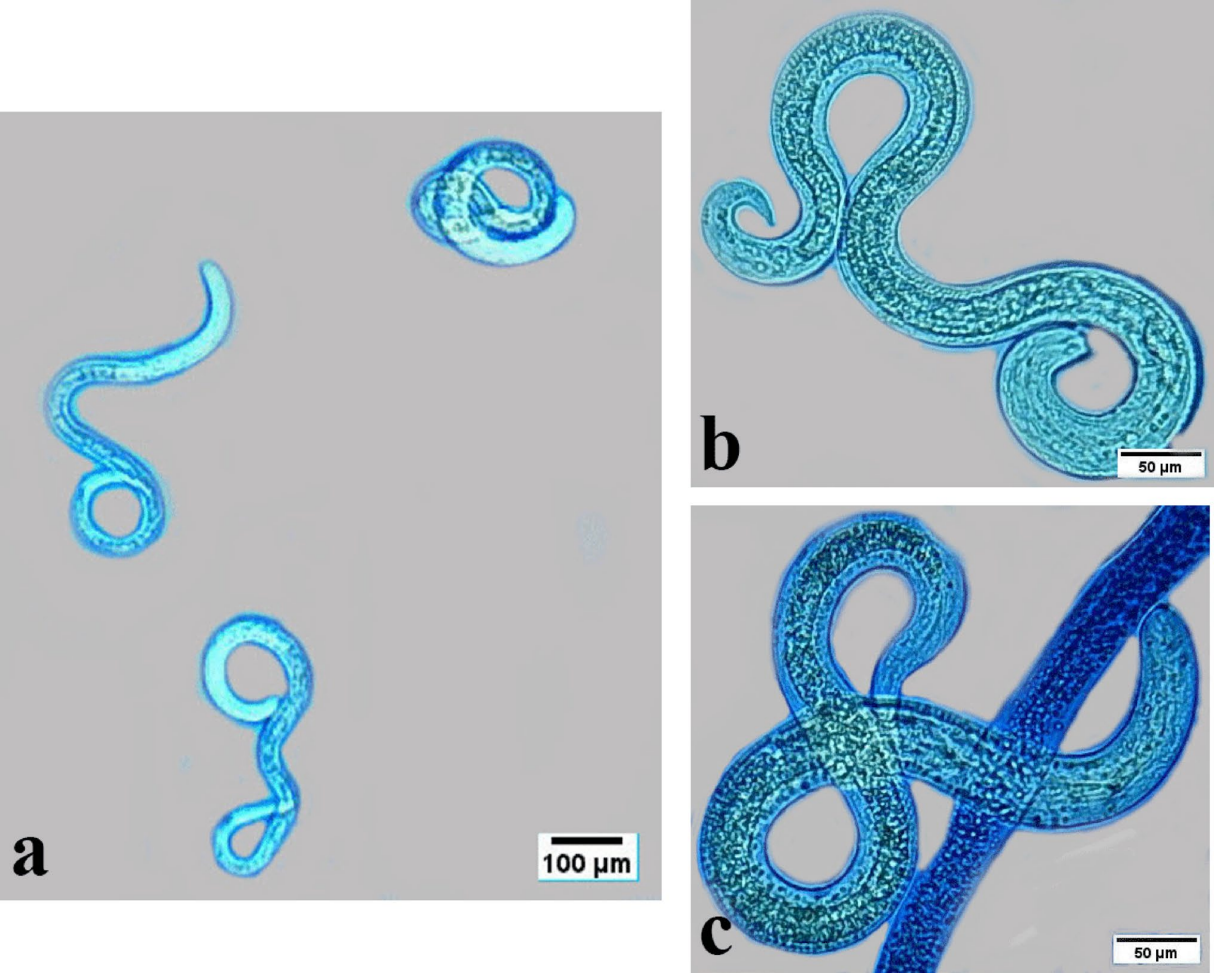
Photomicrographs of *T. vitulorum* larvae from the control group. White arrow live larvae. Red arrow died larvae

*T. vitulorum* larvae from the control group had smooth bodies and were devoid of any structural or morphological changes (Fig. 8). Larvae had a tapering body form and a rounded head. However, it wasn’t easy to distinguish the prominent lips that are characteristic of denticles and ascaridoid nematodes. Larvae obtained from the adult worm that was taken freshly from buffaloes *Bubalus bubalis* exhibited the characteristic coiling behaviour of nematode larvae. Larvae were found to have neither copulatory spicules nor sexual organs. The dead larvae (red arrow) absorbed the bromophenol blue stain, whereas the live larvae (white arrow) did not.

**Fig. 9:**
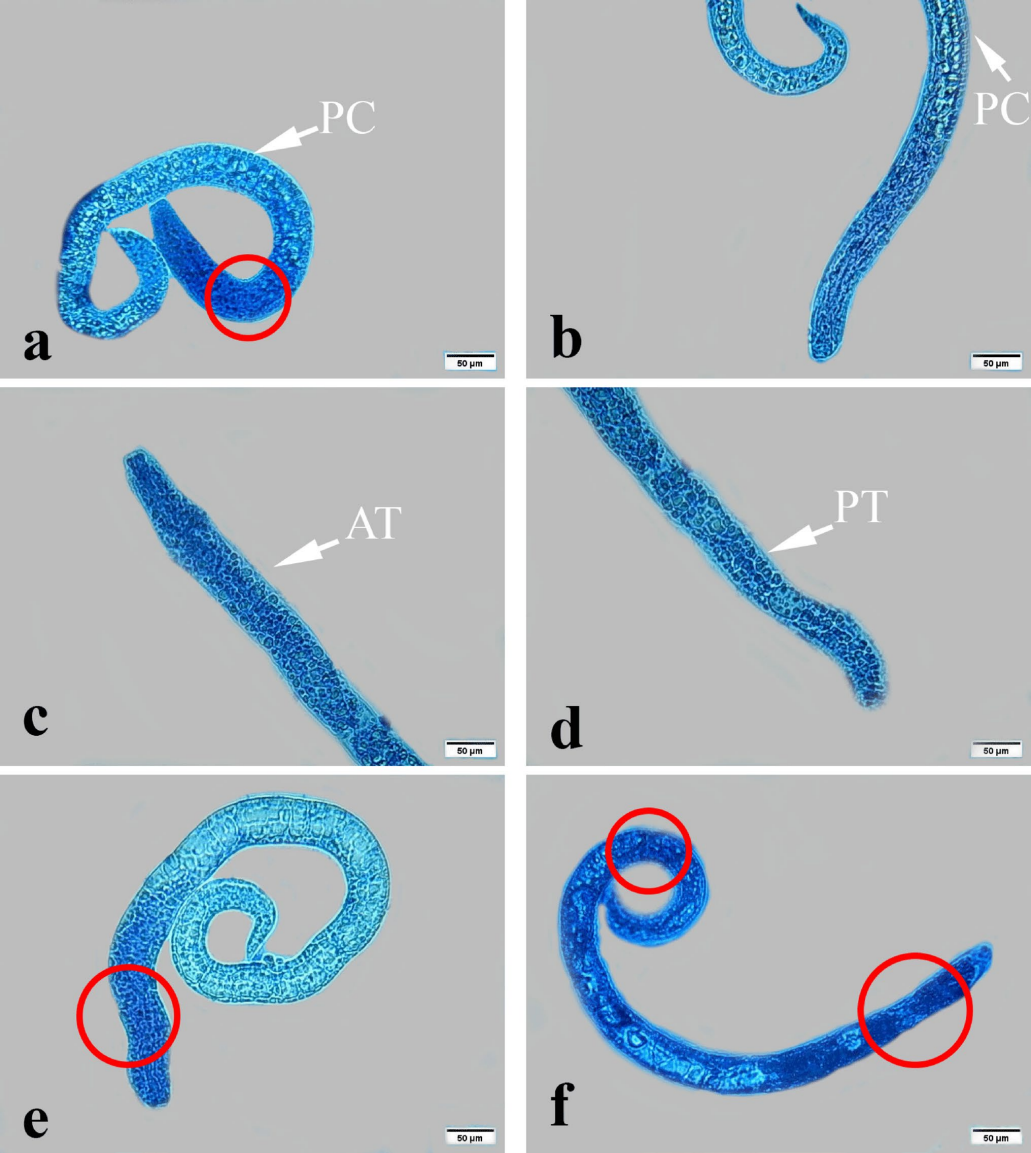
Photomicrographs of exposed *T. vitulorum* larvae to thymol (1000, 2000, 3000, 4000, and 5000 µg/mL). **a** and **b** The larvae exposed to (1000 and 2000 µg/mL) thymol showed a damaged anterior end in a red circle and porous cuticle (PC), **c** and **d** The larvae exposed to (3000 µg/mL) thymol showed an anterior tortuous part (AT) and a posterior tortuous part (PT), **e** The larvae exposed to (4000 µg/mL) thymol showed damage and a cut anterior end in a red circle, **f** The larvae exposed to (5000 µg/mL) thymol showed totally damaged anterior and posterior ends

Light microscopy examination of *T. vitulorum* larvae subjected to thymol (1000, 2000, 3000, 4000, and 5000 µg/mL) showed many serious damages (Fig. 9). The larvae exposed to thymol at concentrations of 1000 and 2000 µg/mL had an altered morphological appearance, with large pores in the cuticle layer and a damaged anterior end (Fig. 9a, b). While exposed to thymol at a dosage of 3000 µg/mL, the larvae lost their nematode-like coiling behaviour. In addition, the anterior and posterior ends lost their normal, rounded, tapering shape and possessed tortuous ends with a severe porous cuticle (Fig. 9c, d). The larvae treated with thymol at a dosage of 4000 µg/mL lost coiling behaviour exclusively in the anterior end, while the posterior end remained alive and unaffected. Larvae treated to thymol at a dosage of 5000 µg/mL completely absorbed the stain and lost coiling behaviour in the anterior end (Fig. 9f).

**Fig. 10:**
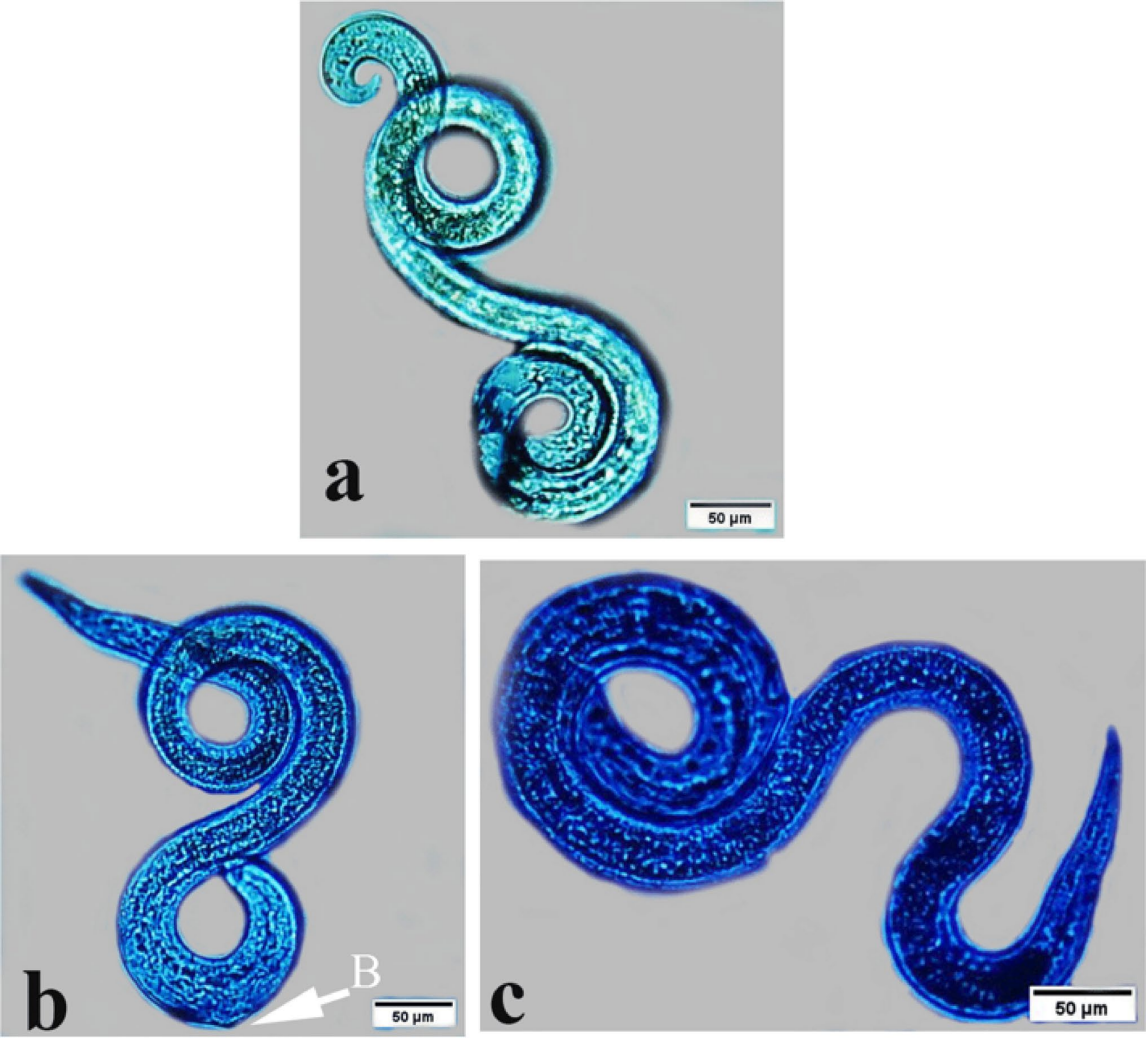

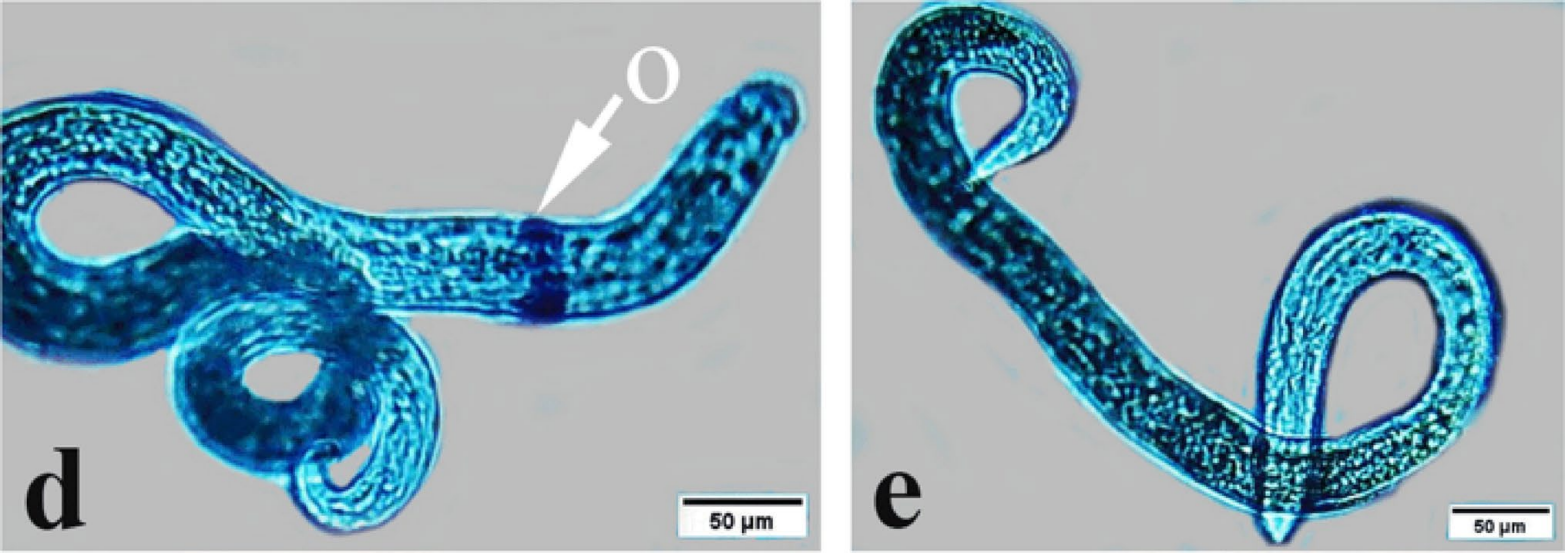
Photomicrographs of exposed *T. vitulorum* larvae to chitosan NPs (1000, 2000, 3000, 4000, and 5000 µg/mL). **a** The larvae exposed to (1000 µg/mL) chitosan NPs showed normal coiling behaviour of nematodes without any deformations. The larvae exposed to (2000 µg/mL) chitosan NPs showed small blebs (B) **c** The larvae exposed to (3000 µg/mL) chitosan NPs showed damage to the anterior end in the red circle, **d** The larvae exposed to (4000 µg/mL) chitosan NPs showed a damaged posterior end with obstructions (O) in the anterior part, **e** The larvae exposed to (5000 µg/mL) chitosan NPs showed damage to the middle part of the body

Light microscopy examination of *T. vitulorum* larvae treated with chitosan NPs (1000, 2000, 3000, 4000, and 5000 µg/mL) showed many morphological defects (Fig. 10). The larvae treated with 1000 µg/mL chitosan NPs exhibited a tapered, rounded head and retained their coiling behaviour. The larvae’s smooth bodies and normal appearance showed no structural or morphological alterations (Fig. 10a). All the larvae exposed to the rest of the concentrations (2000, 3000, 4000, and 5000 µg/mL) had lost their coiling behaviour. In addition, the larvae subjected to chitosan NPs at a concentration of 2000 µg/mL showed some tiny blebs at the anterior end only (Fig. 10b). Furthermore, larvae treated with chitosan NPs at a concentration of 4000 and 5000 µg/mL showed damaged posterior end with obstructions in the anterior part (Fig. 10d, e).

**Fig. 11:**
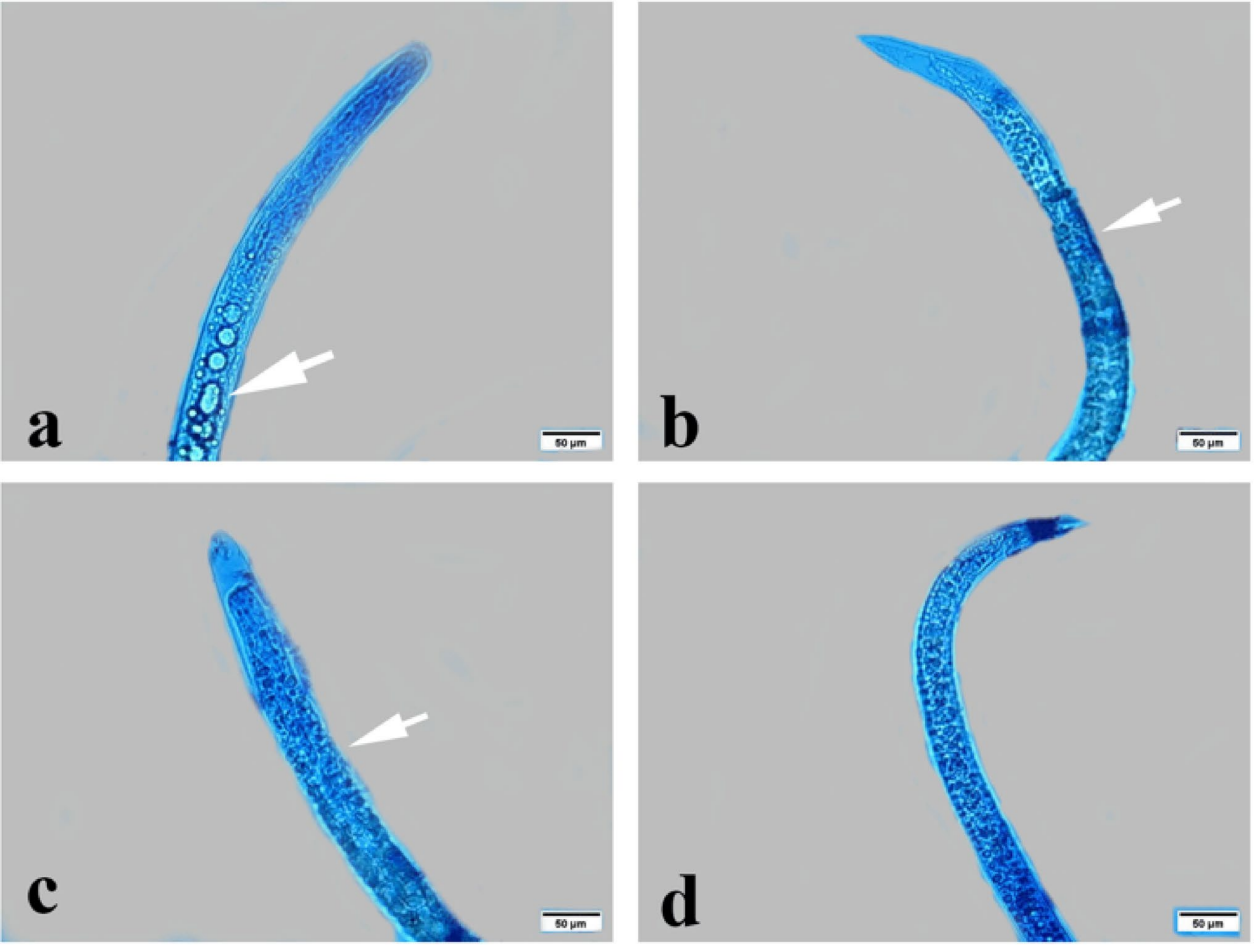

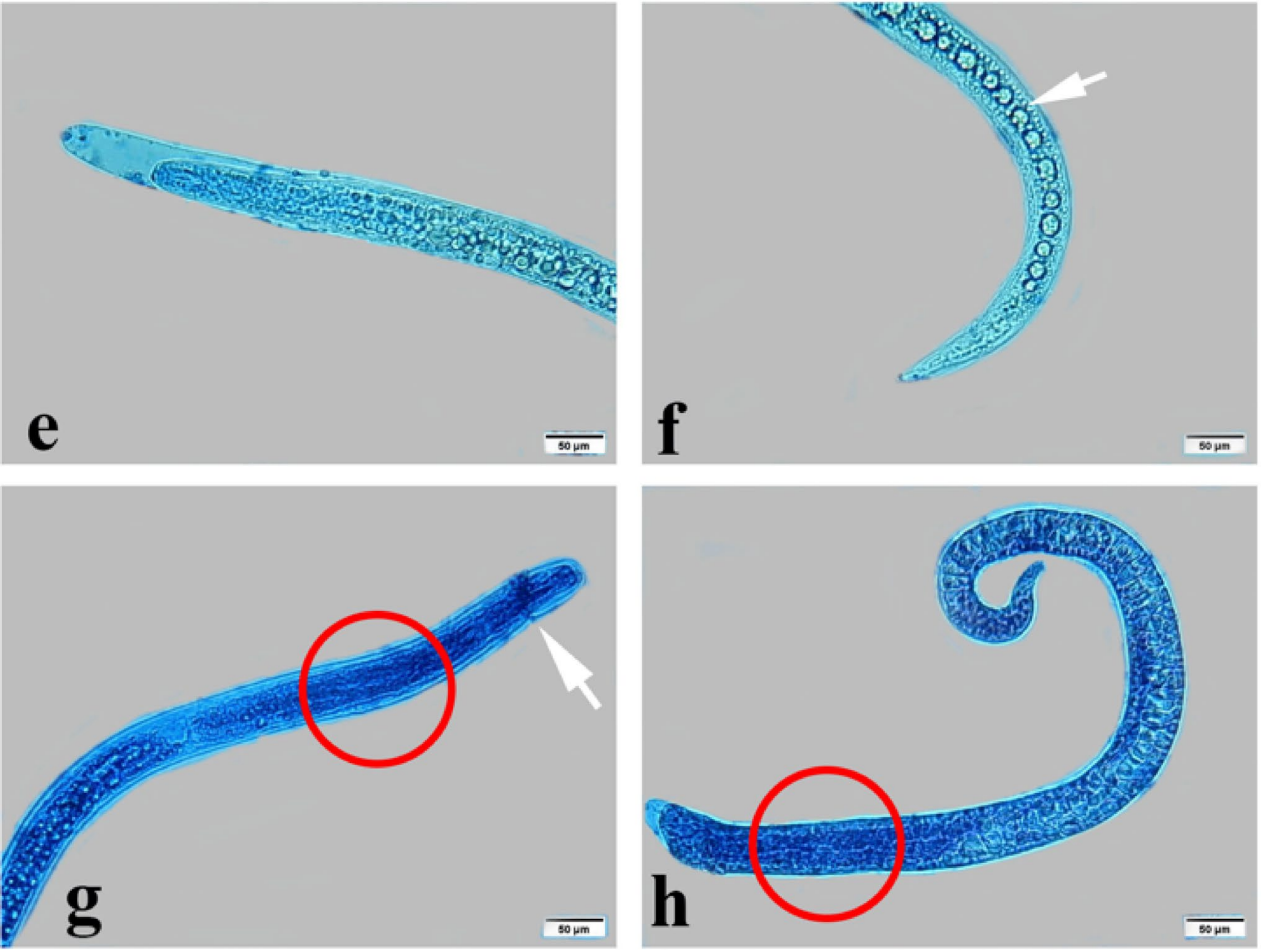
Photomicrographs of exposed *T. vitulorum* larvae to TC NPs (1000, 2000, 3000, 4000, and 5000 µg/mL). **a** The anterior part of larvae exposed to (1000 µg/mL) TC NPs showed a damaged digestive tract in pieces (arrow), **b** The posterior part of larvae exposed to (1000 µg/mL) TC NPs showed a porous cuticle (arrow), **c** and **d** The anterior and posterior parts of larvae exposed to (2000 µg/mL) TC NPs showed porous cuticle, losing coiling behaviour, **e** The larvae exposed to (3000 µg/mL) TC NPs lost the coiling behavior, **f** The larvae exposed to (4000 µg/mL) TC NPs showed a damaged digestive tract: **g** and **h** The larvae exposed to (5000 µg/mL) chitosan NPs showed totally damaged anterior and posterior parts in the red circle with obstruction (arrow)

*T. vitulorum* larvae treated with thymol-loaded chitosan (TC) nanoparticles at concentrations of 1000, 2000, 3000, 4000, and 5000 µg/mL were examined using light microscopy (Fig. 11). All of the larvae that were subjected to all of the different concentrations of TC NPs (1000, 2000, 3000, 4000, and 5000 µg/mL) displayed a straight body and lost their coiling behaviour at the anterior and posterior ends. The stain was absorbed by the anterior end of the larvae that had been treated with 1000 µg/mL of TC NPs, which revealed that the digestive system had been destroyed and appeared as shards along the body. In contrast, the posterior end did not have a sharp point and presented a large number of porous cuticles (Fig. 11a, b). The larvae that were exposed to TC NPs at a concentration of 2000 µg/mL exhibited absorption of the stain due to their porous cuticle. Whereas the larvae that were exposed to TC NPs at concentrations of 3000 and 4000 µg/mL displayed a damaged anterior end and a damaged digestive system that appeared as fragments along the body (Fig. 11e, f). In contrast, the larvae that were exposed to TC NPs at a concentration of 5000 µg/mL were able to absorb the stain completely. Furthermore, the front end was characterized by wrinkles, while the posterior end was not pointed at all and included a large number of porous cuticles (Fig. 11g, h).

### Scanning Electron Microscopy Examination of *T. Vitulorum* Larvae

**Fig. 12:**
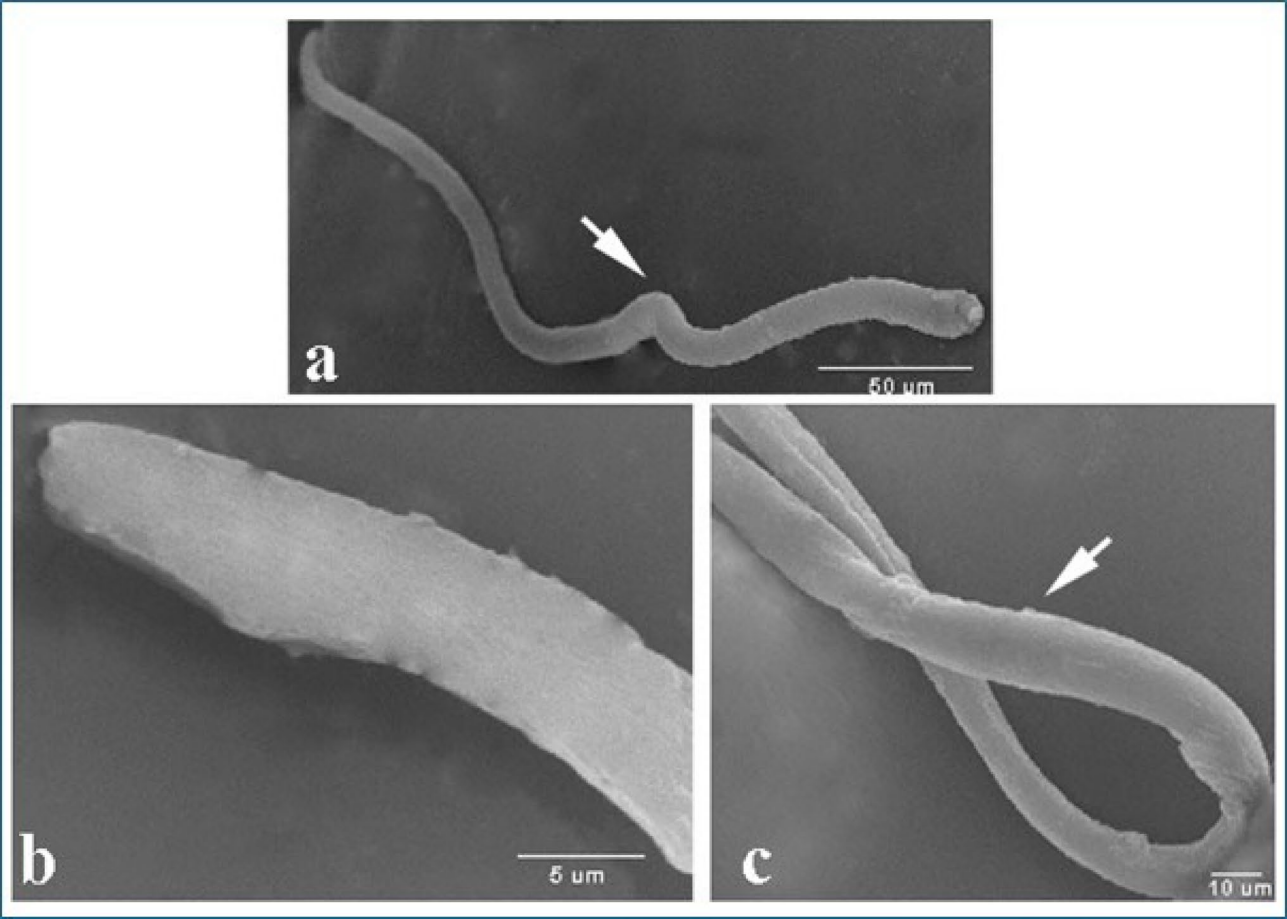
Scanning electron micrographs of *T. vitulorum* larvae from the control group exhibiting normal coiling behavior

The larvae exhibited a smooth body, and compared to the larvae exposed to treatments, no morphological or structural changes were discovered. There was a lack of differentiation in these larvae, which had a head that was rounded and tapering and lacked clarity in the definition of the large lips that are characteristic of ascaridoid nematodes, as well as denticles. Larvae exhibited the distinctive coiling activity that is characteristic of nematode larvae (Fig. 12).

**Fig. 13:**
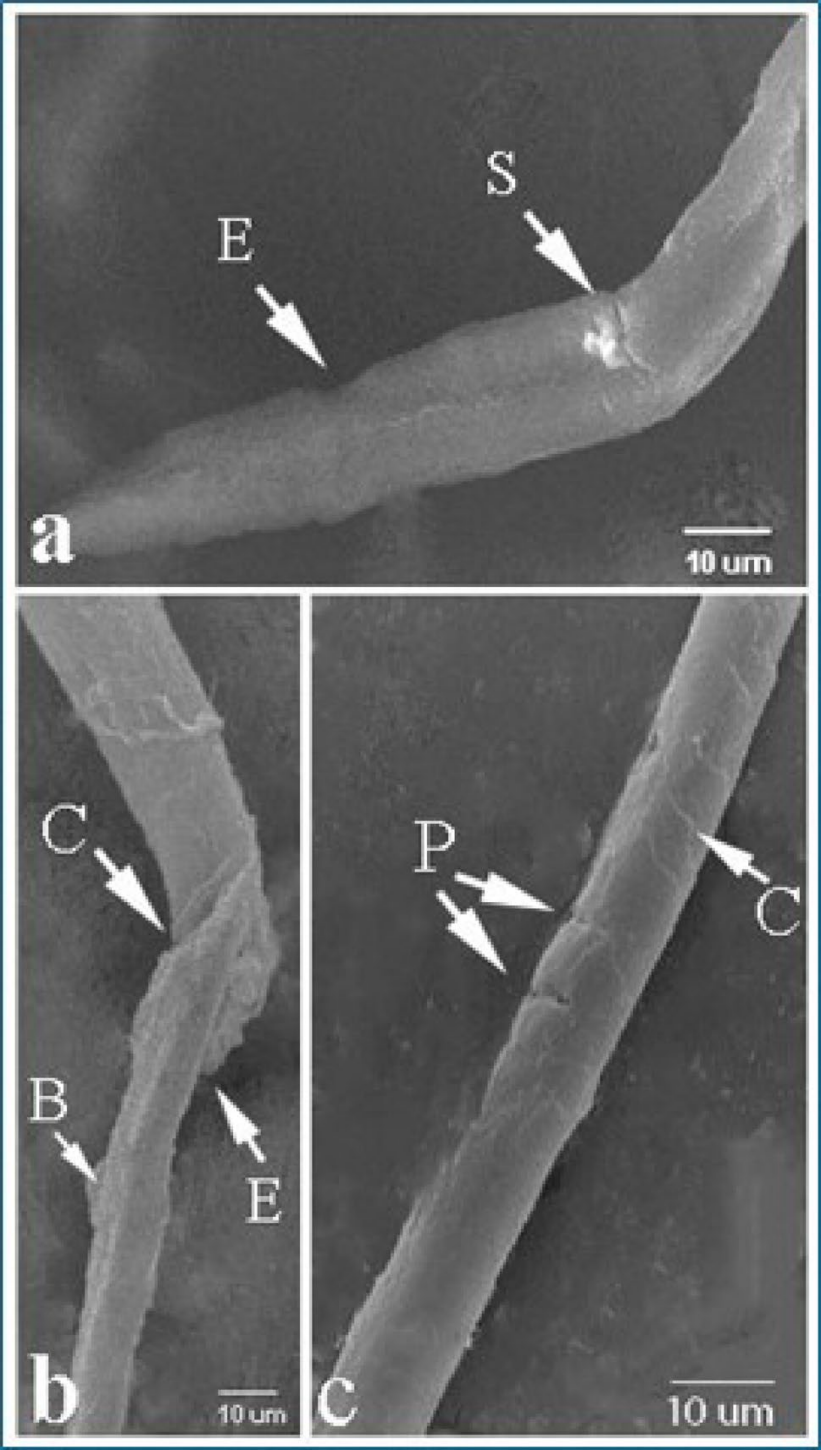
Scanning electron micrographs of *T. vitulorum* larvae exposed to (5000 µg/mL) thymol. **a** Anterior rounded end of larvae exhibited shrinkage (S) and erosional (E) areas, **b** The posterior end of larvae exhibited many blebs (B), cracks and erosions in the cuticle layer, **c** The middle part of the larvae exhibited small pores (P) and cracks (C)

Scanning electron microscopy study of *T. vitulorum* larvae exposed to thymol 5000 µg/mL revealed a change in morphological appearance compared to the control group (Fig. 13). The anterior end of the treated larvae had some erosional regions and shrinking cuticles (Fig. 13a). The posterior end revealed damaged and erosional cuticle with some tiny blebs (Fig. 13b). In contrast, the middle part of the larvae had many cracked areas and small blebs along the body (Fig. 13c).

**Fig. 14:**
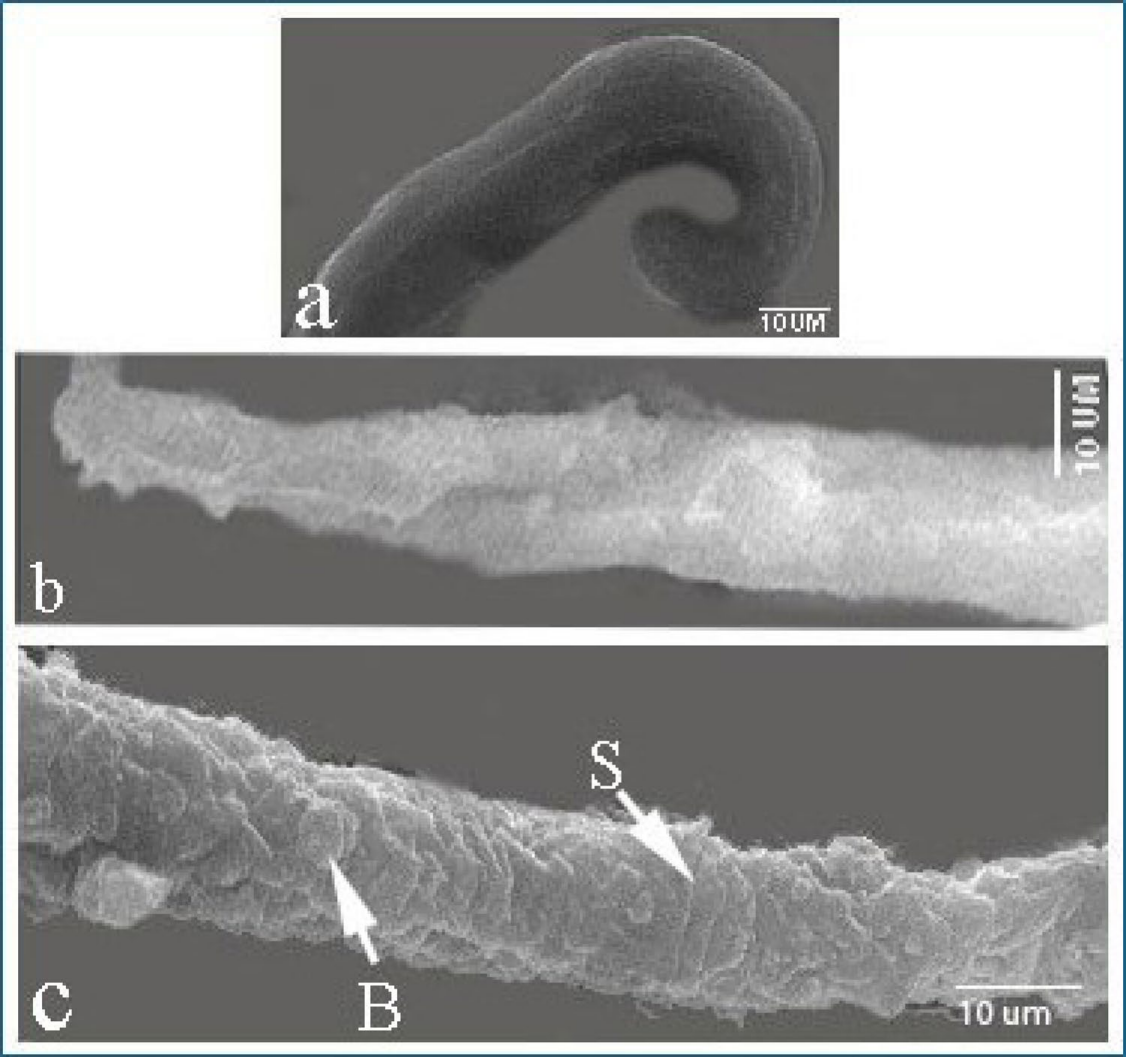
Scanning electron micrographs of *T. vitulorum* larvae exposed to (5000 µg/mL) chitosan NPs. **a** Anterior rounded end of larvae exhibited a smooth cuticle layer, **b** posterior end of larvae, **c** The middle part of the larvae exhibited small pores (P) and shrinkages (S)

SEM examination of *T. vitulorum* larvae treated with chitosan NPs 5000 µg/mL showed many damaged areas compared to the control group (Fig. 14). The anterior end of the treated larvae had smooth cuticle without any deformations. It exhibited the typical coiling behaviour (Fig. 14a). In contrast, the posterior end had many wrinkles, shrinkages and many chitosan depositions on the cuticular layer (Fig. 14b), and the middle part of the larvae had many shrinkages and large blebs along the cuticle (Fig. 14c).

**Fig. 15:**
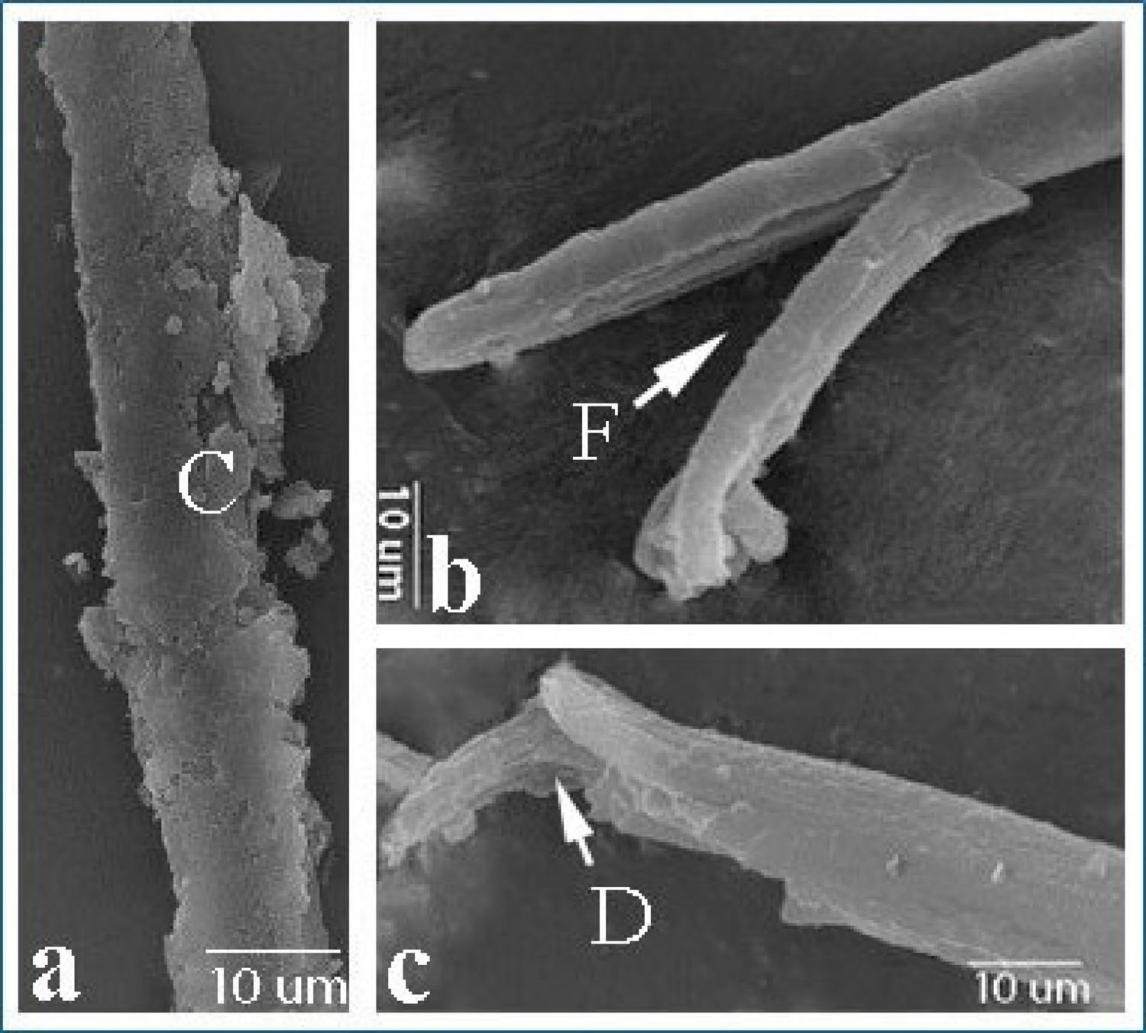
Scanning electron micrographs of *T. vitulorum* larvae exposed to (5000 µg/mL) TC NPs. **a** The Middle part of the larvae exhibited severe cracks (C) in the cuticle layer, **b** The anterior end of larvae exhibited a long furrow (F), **c** The posterior end of larvae exhibited cracks and damage (D)

SEM examination of *T. vitulorum* larvae treated with TC NPs 5000 µg/mL showed many damaged areas compared to the control group (Fig. 15). The larvae were severely damaged in all regions. The anterior portion displayed a deep longitudinal fissure with erosion in the cuticle (Fig. 15b), whilst the posterior end contained several large blebs, severe cracks, and a shattered end in three parts (Fig. 15c.). The central half of the larvae was cracked and smashed, revealing numerous profound fractures and pores in the cuticle layer (Fig. 15a).

## Discussion

Toxocariasis is a worldwide endoparasitic illness caused by *Toxocara vitulorum* that primarily impacts the health of buffaloes and cattle [26]. Infected animals may experience respiratory symptoms, poor thrift, rough hair coat, and diarrhoea or colic [7]. Furthermore, significant adult worm infestations might result in small intestine rupture and host mortality [2]. Piperazine, pyrantel, febantel, and oxfendazole are effective treatments for adult *T. vitulorum*. Pyrantel and levamisole can be used to treat third-stage *T. vitulorum* larvae in the intestine [47].

Although anthelmintic drugs are very successful in most instances, drug resistance has been observed in numerous cases, particularly in *T*. *vitulorum* infection [48]. As a result, new medical treatments with diverse mechanisms are essential, and nanoparticle-based medication formulations can meet that requirement. This study sheds light on the therapeutic efficacy of thymol-loaded chitosan nanoparticles on *Toxocara vitulorum* infected larvae.

A thorough assessment of the various physicochemical properties of the synthesized nanoparticles is necessary to ensure they exhibit the requisite features. The transmission electron microscope is critical for examining the morphological properties of thymol-loaded chitosan nanoparticles. In the current study, TEM investigation revealed the spherical structure of thymol-loaded chitosan nanoparticles, which is in the same line as [31]. This shows a well-organized assembly of nanoparticles measuring 141 nm ± 6 nm.

The average hydrodynamic diameters of the nanoparticles were calculated using the DLS method. Chitosan nanoparticles had the smallest diameter (190 nm), followed by thymol-loaded chitosan nanoparticles (220 nm), which had the largest diameter. This increase in size is expected because the incorporation of thymol into the polymeric matrix causes an increase in overall diameter due to encapsulation or surface contact [49].

All formulations showed homogenous structures based on the polydispersity index values; chitosan nanoparticles had the lowest polydispersity index (PDI) (0.453) [50]. A lower PDI suggests a tighter size distribution, which is preferable for maintaining consistent delivery of drugs and biological interactions. Although values less than 0.3 are normally considered highly monodisperse, PDIs ranging from 0.3 to 0.6 are suitable for nanoparticle compositions intended for biological purposes [51].

The zeta potential measurements revealed the surface charge of the nanoparticles. With zeta likely values of + 51.9 ± 2.5 mV and + 42.4 ± 1.3 mV, respectively, chitosan and thymol-loaded chitosan nanoparticles showed positively charged surfaces. These values imply steady colloidal characteristics for the nanoparticles [52].

Thymol-loaded chitosan nanoparticles have great encapsulation effectiveness (96.6%) and loading capacity (>50%) due to hydrophobic and hydrogen bonding, which improves drug retention in the polymeric matrix. The adjusted drug-to-polymer ratio ensures steady integration without leakage, while the ionic gelation/emulsion process reduces drug loss and improves entrapment. Similar investigations have found high EE for hydrophobic molecules enclosed in chitosan, validating its bioactive chemical stabilization ability [49, 53].

The produced thymol-loaded chitosan nanoparticles exhibited crystalline properties, owing primarily to chitosan’s inherent crystallinity [54]. This crystalline structure is also created by intramolecular hydrogen bonding between polymer chains within the internal structure of the hydrogen. Elevated crystallinity is associated with lower polymer chain mobility and free volume inside the hydrogel matrix. Such structural stiffness impedes solvent molecule penetration and slows drug diffusion across the matrix. As a result, crystalline areas operate as barriers to water ingress and drug transport, lowering the overall disintegration rate of the embedded medication [55]. Furthermore, these crystalline domains increase crosslink density, which improves thymol’s mechanical stability and swelling resistance under physiological conditions.

Free radicals are generated by a variety of regular biological processes, such as aerobic metabolism and pathogenic defence mechanisms, in relation to oxidative stress markers. Reactive oxygen species (ROS) are a subset of free radicals that contain oxygen, including superoxide anion (O2−), hydrogen peroxide (H2O2), and hydroxyl radical (HO•) ( [56]. Lipid peroxidation (LP) is a multifaceted chain reaction that results in the degradation of unsaturated fatty acids in cell membranes as a result of the influence of free oxygen radicals. Consequently, LP is the most accurate indicator of ROS levels that resulted in systemic biological injury [57]. The degree of LP and the level of ROS are indirectly evaluated by the estimation of malondialdehyde (MDA) level, as MDA is the final product of lipid peroxidation [58].

The present study showed an elevation in MDA level in the exposed larvae to thymol and chitosan NPs (1000, 2000, 3000, 4000, 5000 µg/mL), while the level of MDA was extremely high after exposing the larvae to TC NPs (1000, 2000, 3000, 4000, 5000 µg/mL), as compared to the control group. In the context of the reports of Ali et al. [8], the substantial increase in MDA levels observed in the present study may indicate that lipid peroxidation was enhanced as a result of the exposure to NPs, which is indicative of free radical-mediated larval cell membrane damage.

Nitric oxide (NO) is a significant reactive nitrogen species in biological systems. NO can react with multiple oxidative molecules, including molecular oxygen (ROS), transition metals, and thiols, to produce a variety of reactive nitrogen species [59]. NO is required for a range of helminthes’ biological functions, including muscle relaxation, and it acts as a modulator of neuropeptide activity in ionic channels [60]. The study found that exposure to thymol and chitosan NPs increased NO levels in larvae. Still, exposure to TC NPs resulted in much higher levels compared to the control groups. This aligns with prior studies indicating that nitric oxide (NO) is crucial in the detoxification of reactive oxygen species generated by nanoparticle exposure in larvae and acts as a deoxygenase, utilizing NO to remove oxygen [61].

Free radicals are neutralized by antioxidants, which prevent oxidative stress and cellular damage [62]. GSH, alpha-lipoic acid, SOD, CAT, and GSH peroxidase are endogenous, and exogenous sources include vitamin C, vitamin E, beta-carotene, selenium, bioflavonoids, and N-acetyl cysteine (NAC) [63]. Reduced glutathione (GSH) is the most prevalent non-protein thiol molecule and a key component of detoxification pathways [64]. Catalase (CAT) is an essential element of the antioxidant defense mechanism [56]. CAT facilitates the breakdown of hydrogen peroxide into molecular oxygen and water [65].

In contrast to the control group, the current investigation demonstrated that the GSH and CAT levels were significantly greater in the larvae subjected to TC NPs than in the larvae exposed to thymol and chitosan NPs.

The increased free radical trapping efficacy that may have contributed to the increased production of non-enzymatic antioxidant GSH following TC NPs exposure [66]. Treatment with nanoparticles enhances transcription factor Nrf2 expression, which influences the transcription of genes encoding for cyto-protective proteins, such as CAT, resulting in increased CAT activity [67].

The investigation of larvae using light microscopy and scanning electron microscopy from several perspectives is crucial for elucidating morphological alterations. The findings derived from the LM and SEM in this study concern the in vitro treatment of *T. vitulorum* with TC NPs and its impact on the anterior, middle, and posterior regions of the larvae. The shrinkage and wrinkling of the anterior and posterior sections in the treated larvae with TC NPs align with the findings of Bahaaeldine et al. (2022) [68], who demonstrated the stress and toxic effects of NPs on the normal morphology of nematodes such as *Ascaris* sp. and *Toxocara* sp., potentially due to the direct interaction and absorption of the NPs by the worms’ bodies.

The cuticle is a resilient extracellular layer that safeguards the worm from environmental factors and preserves the organism’s morphology and structural integrity [46]. The surface topography of the exposed larvae to TC NPs demonstrated significant damage to the cuticle, characterized by the appearance of many vesicles, holes, sloughing, and blebs. This aligns with the findings of Ahsan et al. [69], which demonstrated significant damage to the worm’s cuticle following treatment with nickel oxide nanoparticles. One of the defining consequences of nanoparticles was the degradation of the worm’s cuticle. Nanoparticles influence the cuticle by binding released positive ions to the negatively charged cell membrane, hence compromising membrane integrity. NP ions may also bind to the membrane wall, creating pores and openings that facilitate their penetration into the organism. Also, the blebs and vesicles represent the larvae’s effort to restore the compromised surface membrane in reaction to nano activity [46].

## Conclusion

This study has shown the great potential of using nanotechnology in the presentation of effective control of important nematode larvae, and further work is recommended to determine if the important observations made in vitro can be translated into in vivo situations.

## Data Availability

No datasets were generated or analysed during the current study.
